# PTEN Inhibits Inflammatory Bone Loss in Ligature-Induced Periodontitis via IL1 and TNF-*α*

**DOI:** 10.1155/2019/6712591

**Published:** 2019-11-30

**Authors:** Chuanyun Fu, Zhimin Wei, Dongsheng Zhang

**Affiliations:** ^1^Department of Orthodontics, Shandong Provincial Hospital Affiliated to Shandong University, Jinan, Shandong 250021, China; ^2^Department of Oral and Maxillofacial Surgery, Shandong Provincial Hospital Affiliated to Shandong University, Jinan, Shandong 250021, China; ^3^Key Laboratory of Shandong Provincial Hospital, Jinan, Shandong 250021, China

## Abstract

Phosphatase and tensin homolog (PTEN) is a critical regulator of tumorigenesis and bone remodeling, which is also found expressed in the periodontal tissues. Periodontitis is one of the most common oral diseases and associated with alveolar bone resorption and tooth loosening in adults. However, the functional relevance of PTEN in periodontitis remains unclear. Here, we report that PTEN plays an essential role in periodontitis. The in vivo results of our study showed a significant decrease of PTEN in the ligature-induced mouse periodontitis model. The function of PTEN in the macrophages was shown to be associated with inflammatory factors interleukin 1 (IL1) and tumor necrosis factor (TNF-*α*) by using overexpression and silence methods. Further mechanistic studies indicated lack of PTEN-activated IL1 and TNF-*α*, which increased the number of osteoclasts and led to alveolar bone erosion and loss. Moreover, PTEN nanoparticles could directly inhibit the inflammatory process and bone erosion, suggesting a controlling role of PTEN during bone remodeling. All these data identified the novel function of PTEN as a key factor in periodontitis and bone remodeling.

## 1. Introduction

Inflammatory osteolysis is a critical pathological feature of clinical inflammatory diseases such as periodontitis which is one of the most common oral diseases and associated with alveolar bone resorption and tooth loosening in adults [1]. The therapeutics for periodontitis are intended to block inflammation locally, but not enough for inflammatory bone destruction [2]. However, many patients still suffer from bone loss under the anti-inflammation treatments [3]. Therefore, more effective and economic treatments to reduce the inflammation and bone destruction are urgently needed. In the pathogenesis of periodontitis, osteolysis is predominantly caused by increased osteoclasts number and activity [3, 4]. The most determined cytokine involved in osteoclastogenesis is the receptor activator of nuclear factor *κ*B ligand (RANKL) [5]. Presence of RANKL could be modulated by several proinflammatory cytokines such as tumor necrosis factor (TNF-*α*), interleukin 1 (IL1), and interleukin 6 (IL6) [6]. Thus, an ideal therapeutic strategy to prevent inflammatory bone destruction is to suppress proinflammatory cytokines activation during osteoclastogenesis.

The phosphatase and tensin homolog deleted on chromosome 10 (PTEN) is a specific tumor suppressor gene which can efficiently limit phosphoinositide 3-kinase (PI3K) activity and downstream serine/threonine kinase Akt (also known as protein kinase B or PKB) signaling [7]. Several studies indicate that the PI3K/PTEN pathway regulates the inflammatory response [8, 9]. The nuclear factor kappa-light-chain-enhancer of activated B cells (NF-*κ*B) pathway is the classical proinflammatory signaling pathway based on the role of NF-*κ*B in proinflammatory gene expressions. The study has shown that Toll-like receptor (TLR)-induced PI3K /Akt activation is phosphorylated, thereby inhibiting downstream glycogen synthase kinase (GSK) 3*β*, leading to a diminished expression of NF-*κ*B-driven proinflammatory genes in monocytes [10]. PTEN also plays an important role in varieties of cellular regulation, including survival, apoptosis, autophagy, migration, and proliferation [11]. However, its role during inflammatory bone loss remains unclear.

In the present study, ligature-induced periodontitis in wild type (C57BL/6J) mice were used as in vivo models and RAW264.7 cells were used for in vitro studies. The protective effects of PTEN on ligature-induced periodontitis and the underlying mechanisms associated with inflammatory factors regulation were investigated. Our study provides a novel insight into understanding the protective effects of PTEN on inflammation and bone remodeling in periodontitis and proposes that PTEN can be used as an adjuvant therapy for inflammatory diseases.

## 2. Materials and Methods

### 2.1. Animals

All mice were maintained in C57BL/6J background and housed under pathogen-free conditions with a 12-hour light/dark cycle and fed regular chow and sterile water throughout the experimental period. Our study was approved by the Ethics Committee of Shandong Provincial Hospital affiliated to Shandong University. All experiments were performed according to the Regulations and Guidelines approved by the Shandong Provincial Hospital Animal Care and Use Committee.

### 2.2. Generation of Ligature-Induced Periodontitis Model

Ligature-induced periodontitis model was performed as described previously with slight modification [12]. A 5-0 silk ligature (Roboz Surgical Instrument Co., MD, USA) was tied gently around the left maxillary second molar under anesthesia to induce periodontitis, which was maintained for 10 days, and the contralateral molar was served as a control. The ligatures remained in place in all mice throughout the experimental period, monitored every 3 days and retied if the ligature was loose or gone. Furthermore, if the ligature was not present at the termination of the experiment, the sample was removed from the analysis.

### 2.3. Nanoparticle Therapy

To prepare nanoparticle for in vivo treatment, 10 *μ*g of recombinant PTEN plasmid (pcDNA3.1-PTEN) or pcDNA3.1 (control) was mixed with 20 *μ*l of nanoparticle in vivo DNA transfection reagent (Engreen Biosystem Co. Ltd., Beijing, China) according to previous studies and the manufacturer's protocol [13, 14]. Mice (10 weeks old) were given local injection of nanoparticles containing 10 *μ*g plasmid at the left maxillary second molar periodontal tissues every 3 to 4 days during the two weeks of therapy.

### 2.4. Histology and Histological Scoring

Histological studies were performed as previously described [15]. Briefly, samples were decalcified in 10% ethylenediaminetetraacetic acid (EDTA) for 4 weeks. Specimens were optimal cutting temperature compound- (OCT-) embedded, cut into 7 *μ*m-thick sections in the coronal plane using a microtome, and stained with hematoxylin and eosin (H&E) (Abcam, UK). In addition, samples were further stained with tartrate-resistant acid phosphatase (TRAP) (Sigma-Aldrich, MO, USA) to evaluate the number of osteoclasts [16].

All specimens were blindly analyzed by an experienced examiner and scored for inflammation and bone destruction [17–19]: Score 0: no inflammation and bone destruction, normal gingiva; Score 1: mild inflammation, sparse mononuclear cells, osteoclast activation; Score 2: moderate inflammation, monocyte infiltration, and/or sparse eosinophils or neutrophils, osteoclasts lacunas; Score 3: severe inflammation, eosinophils or polymorphonuclear neutrophils infiltration, with abscess areas, signs of bone erosion; Score 4: severe inflammation with severe alveolar bone erosion and resorption.

### 2.5. Plasmid or siRNA Transfection

Plasmids were constructed by cloning the PTEN gene open-reading frame (ORF) into pcDNA3.1-Flag. For siRNA transfection, RAW264.7 cells were transfected with siRNA targeting PTEN (#1 sense strand: CGTTAGCAGAAACAAAAGGAG and #2 sense strand: GATCTTGACCAATGGCTAA) using Lipofectamine™ 2000 reagent (Thermo Fisher Scientific, USA) according to the manufacturer's protocol. A final concentration of 100 nM siRNA was used. After transfection for 48 hours, quantitative real-time PCR analysis was used to assess the mRNA content.

### 2.6. RNA Analysis

RNA analysis was performed as described with a minor modification [20]. Message RNA from mouse molar periodontal tissues was isolated using Trizol reagent (Life Technologies, USA). 2 *μ*g of RNA was reverse transcribed into complementary deoxyribonucleic acid (cDNA) using the Reverse Transcription Kit (TAKARA, Japan). The reaction mixture we prepared contained primers, the cDNA template, and the double stranded DNA-specific dye SYBR Mix (Bimake, USA). The primers' sequences are shown in Table 1.

### 2.7. Statistical Analysis

All data were expressed as the mean ± SEM (*n* ≥ 5). To determine the difference between two groups, a two-tailed Student's *t*-test was performed. To compare more than two groups, a two-way analysis of variance (ANOVA) followed by a Bonferroni post hoc test with a 95% confidence interval (Graphpad Prism, USA) was used. *p* values <0.05 were considered to be statistically significant.

## 3. Results

### 3.1. Ligature-Induced Periodontitis and Inflammatory Bone Loss in Mice

**Figure 1:**
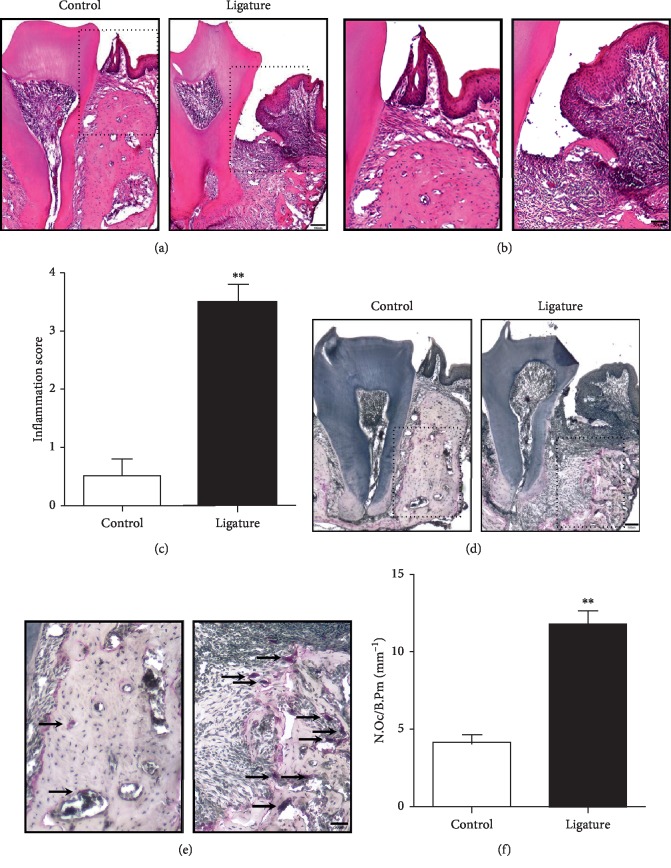
Ligature induced the periodontitis and inflammatory bone loss. (a) Histologic changes in periodontal tissue were monitored by hematoxylin and eosin (H&E) staining. Noticeable inflammatory reaction and damage to organizational structures were observed in ligature-induced periodontitis. Scale bars, 100 *μ*m. (b) Higher magnification of (a). Scale bars, 50 *μ*m. (c) Quantitative analysis of inflammation score of maxillary second molar periodontal tissues. Values are the mean ± SEM. Asterisks (^*∗∗*^) denote significant differences (*p* < 0.01), *n* = 5 biological replicates. (d) TRAP staining showed a significant increase of TRAP-positive multinucleated osteoclasts in mouse maxilla in the ligature-induced periodontitis group. Scale bars, 100 *μ*m. (e) Higher magnification of (d). Scale bars, 50 *μ*m. (f) Quantitative analysis of TRAP-positive multinucleated osteoclasts showed a significantly increased number of osteoclasts in the ligature-induced periodontitis group compared to unligated group. N.Oc/B.Pm (mm^−1^): number of osteoclasts per bone perimeter. Values are the mean ± SEM. ^*∗∗*^*p* < 0.01, *n* = 5 biological replicates.

To assess the changes in inflammation and bone mass, histological analysis was performed. Ligature-induced periodontitis mice showed a significant gingival swelling, a greater soft tissue thickness, and severe bone resorption compared to the unligated group (Figure 1(a)). In addition, a wide range of inflammatory cells were shown around the root of the second molar (Figures 1(a) and 1(b)) and the inflammation score was significantly increased in ligature-induced periodontitis mice (Figure 1(c)). Ten days after ligature, there was a significant difference in bone mass between the ligature-induced periodontitis group and the unligated group. The bone loss around the ligature was significantly increased in mice compared to unligated controls (Figure 1(a)). To assess osteoclast activity, samples were further analyzed by TRAP staining (Figures 1(d) and 1(e)). The result showed that TRAP-positive multinucleated cells increased significantly in the ligature-induced periodontitis group compared to the unligated group (Figure 1(f)).

### 3.2. Gingival mRNA Expressions of PTEN and Inflammatory Cytokines

**Figure 2:**
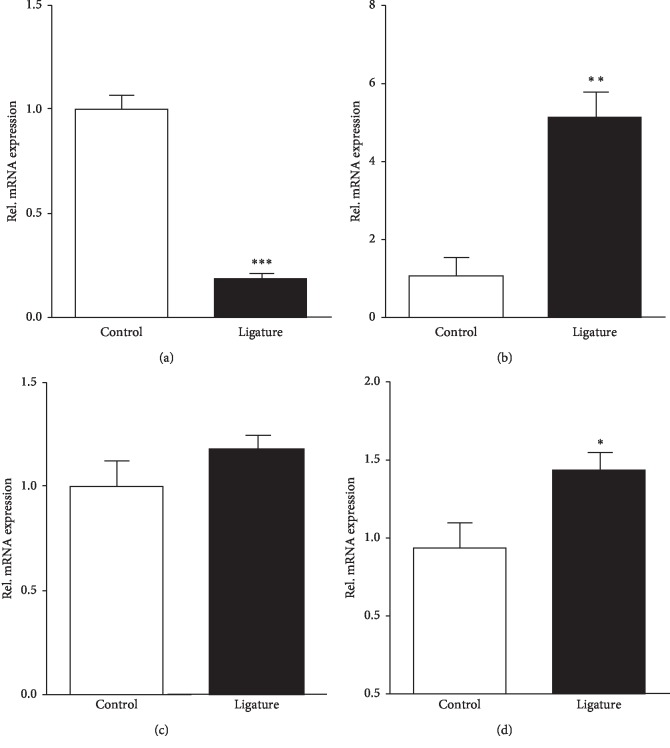
PTEN mRNA were reduced and inflammatory factor mRNA was increased by ligature. (a) Quantitative real-time PCR analysis of PTEN mRNA transcripts from ligature-induced and control mice. Each sample was standardized to GAPDH levels and run in triplicate. Data represent PTEN expression in periodontal tissue relative to control. *n* = 5 biological replicates. Inflammatory factors (b) IL1, (c) IL6, and (d) TNF-*α* were measured as described in Materials and Methods from periodontal tissues. *n* = 5 biological replicates. Values are the mean ± SEM. ^*∗*^*p* < 0.05; ^*∗∗*^*p* < 0.01; *n* = 5 biological replicates.

**Figure 3:**
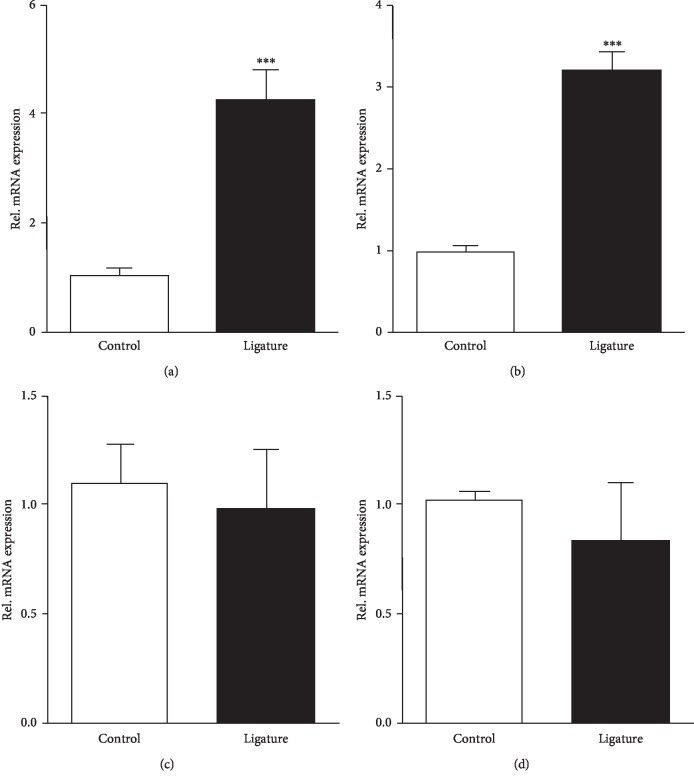
Ligature-induced periodontitis enhanced bone erosion but not bone formation. Levels of expression of mRNA for (a) osteoclast TRAP and (b) cathepsin K in tissue extracted from periodontal tissue obtained on day 10 from control and ligature mice. Data were normalized for GAPDH expression. Values are the mean ± SEM. ^*∗*^*p* < 0.05; ^*∗∗∗*^*p* < 0.001; *n* = 5 biological replicates. Levels of expression of mRNA for the (c) osteoblast marker OC and (d) ALP in tissue extracts from periodontal tissue obtained on day 10 from control and ligature mice as in (a). Values are the mean and SEM of 5 mice per group.

PTEN, as a regulator of the mitogen-activated protein kinase (MAPK) pathway and inflammatory cytokines, was found decreased in ligature-induced periodontitis (Figure 2(a)). And the inflammatory cytokines such as IL1, IL6, and TNF-*α* mRNA expression levels were increased in the ligature-induced periodontitis group, especially the expression of IL1 and TNF-*α* (Figures 2(b)–2(d)). Inflammation was shown to cause excessive bone resorption as well as impaired bone formation. Thus, we determined the bone formation gene markers, osteocalcin (OC) and alkaline phosphatase (ALP), and bone erosion markers, tartrate-resistant acid phosphatase (TRAP) and cathepsin K (Figure 3). The results showed that the gene expressions of bone erosion makers TRAP and cathepsin K were significantly increased in the ligature-induced periodontitis (Figures 3(a) and 3(b)), rather than bone formation markers OC and ALP (Figures 3(c), and 3(d)).

### 3.3. PTEN Regulated the Expression of Inflammatory Cytokines

**Figure 4:**
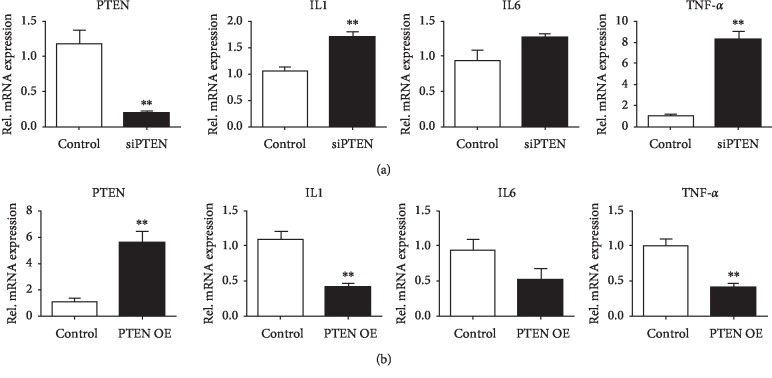
PTEN could positively regulate inflammatory factors. PTEN affects the expression of inflammatory factors. RAW264.7 cells were treated with control siRNA (MOCK) and siPTEN (a) or transfected with pcDNA3.1 (MOCK) and pcDNA3.1-PTEN plasmids (PTEN overexpression; PTEN OE) (b) for 48 h and used to measure PTEN, IL1, IL6, and TNF-*α* mRNA levels by quantitative real-time PCR. The results showed that overexpression of PTEN inhibits inflammatory factors, while knockdown of PTEN promotes inflammatory factors (IL1, IL6, and TNF-*α*). Values are the mean ± SEM. ^*∗∗*^*p* < 0.01; *n* = 5 biological replicates.

Macrophages play important roles in the pathogenesis of periodontitis by regulating the immune response and regulating tissue repair and bone loss [21, 22]. We first silence the PTEN expression in RAW264.7 cells through siRNA and found the same results as those of in vivo experiment; the expressions of inflammatory cytokines IL1 and TNF-*α* were significantly increased (Figure 4(a)). To further confirm this, we then forced overexpression of PTEN in RAW264.7 cells. The results showed that overexpression of PTEN reduced the expression of IL1 and TNF-*α* (Figure 4(b)).

### 3.4. Overexpression of PTEN Inhibits the Inflammation In Vivo

**Figure 5:**
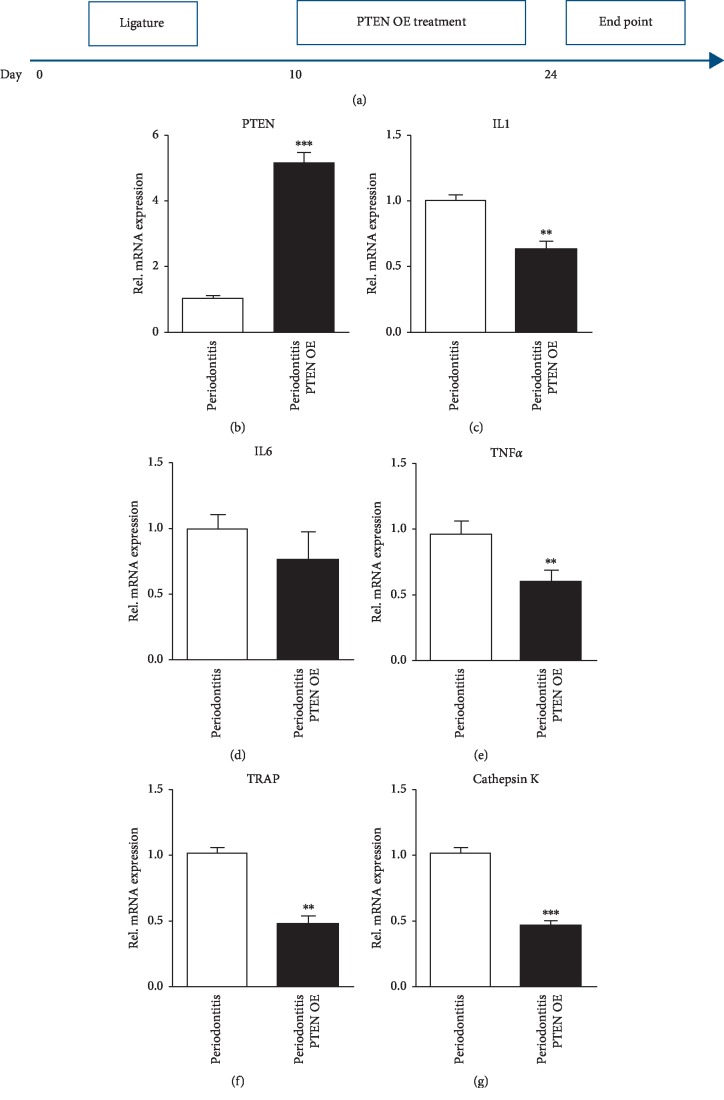
Forced overexpression of PTEN could inhibit inflammation and bone erosion. (a) Schematic figure shows PTEN nanoparticles (10 *μ*g PTEN plasmids with nanoparticles) treated with the ligature on day 10 for two weeks, three times per week. (b) Levels of expression of mRNA for PTEN in periodontal tissue extracts obtained on day 24 from ligature and ligature with PTEN nanoparticle treatment mice. Data were normalized for GAPDH expression. Values are the mean ± SEM. ^*∗∗∗*^*p* < 0.001; *n* = 5 biological replicates. Relative mRNA expression levels for the inflammatory marker genes (c) IL1, (d) IL6, and (e) TNF-*α* in tissue extracts from periodontal tissue obtained as in (b). Values are the mean ± SEM. ^*∗∗*^*p* < 0.01; *n* = 5 biological replicates. Relative mRNA expression levels for the osteoclast marker genes (f) TRAP and (g) cathepsin K in tissue extracts from periodontal tissue obtained as in (b). Note that osteoclast marker genes decreased when treated with PTEN nanoparticles. Values are the mean ± SEM. ^*∗∗*^*p* < 0.01; ^*∗∗∗*^*p* < 0.001; *n* = 5 biological replicates.

To further study the anti-inflammatory effects of PTEN, we made PTEN nanoparticles by mixing the 10 *μ*g PTEN plasmids with 20 *μ*l nanoparticles and injected the mixture into the gingiva three times/week for two weeks (Figure 5(a)). After injection, we found that PTEN gene expression was increased (Figure 5(b)) while the inflammatory factors such as IL1, IL6, and TNF-*α* were decreased, especially the expression of IL1 and TNF-*α* (Figures 5(c)–5(e)). Moreover, osteoclast markers TRAP and cathepsin K were downregulated after exogenous PTEN injection (Figures 5(f) and 5(g)). Conclusively, our results showed that nanoparticle-packaged PTEN can alleviate inflammatory osteolysis.

## 4. Discussion

Our in vitro and in vivo data highlight the anti-inflammatory role of PTEN presentation. The schematic structure of the predicted PTEN protein has been reported [23]. The phosphoinositide 3-kinases can be inhibited by the protein and lipid phosphatase activity of PTEN [24]. Here, we found the gene expression level of PTEN is responsible for inflammation regulation. However, we cannot neglect the effect of protein and its postmodification; thus, further study will focus on the protein and modification level.

In our study, inhibition of PTEN expression with siRNA results in upregulation of IL1 and TNF-*α*, whereas the expression of IL6 was not significantly altered, suggesting that PTEN may exert anti-inflammatory effects in mouse periodontitis by modulating the expression of IL1 and TNF-*α*. NF-*κ*B is generally considered to be the major regulatory pathway of genes encoding proinflammatory proteins including IL1, IL6, and TNF-*α* [25]. The expression of TNF-stimulated NF-*κ*B-dependent genes can be blocked by PTEN [26], which supports our hypothesis that PTEN can play a role in regulating the expression of proinflammatory proteins IL1 and TNF-*α*. In addition, PTEN can regulate NF-*κ*B via the PI3K/Akt pathway [9]. Under physiological conditions, PI3K/Akt/PTEN signaling plays a key role in inflammation [27]. As the common downstream molecules of NF-*κ*B pathway, IL1, IL6, and TNF-*α* play a role in maintaining the cell homeostasis [28–30], further suggesting that PTEN may regulate the inflammatory response through the PI3K/Akt/NF-*κ*B pathway.

PTEN enforced overexpression in RAW 264.7 cells inhibited inflammation and osteoclast in our mouse model, indicating that PTEN may act as a critical regulator between inflammation and bone remodeling. Some relative studies have shown that downregulating PTEN increased phosphorylation of Akt which can promote osteoclastogenesis [31–33]. We herein found that PTEN can regulate osteoclast activity through inflammatory factors, particularly IL1.

Numerous studies have demonstrated that IL1 can stimulate osteoclast differentiation and activation [34–37]. In addition, inhibiting IL1 significantly reduces bone erosions and cartilage degradation, whereas blocking TNF-*α* decreases inflammation [35, 38, 39]. IL1 may increase multinucleated cell formation through direct stimulation [40]. IL1 is capable of inducing CSF-GM production and can stimulate CSF-GM production in the bone marrow. CSF-GM stimulates the proliferation and differentiation of CFU-GM, the probable progenitor for osteoclast [41, 42]. We therefore speculate that PTEN regulate the bone remodeling mainly through activation of IL1. However, no significant changes were found in osteoblast marker genes, suggesting that PTEN primarily regulates osteoclasts rather than osteoblasts during bone remodeling.

## 5. Conclusions

In summary, we report here for the first time that activation of PTEN is necessary for inhibition of inflammation and alveolar bone loss, which suppresses both processes via inhibiting the IL1 and TNF-*α* pathways in periodontitis.

## Figures and Tables

**Table 1:** List of primers sequence for qPCR.

Target (Genbank no.)	Forward primer (5′–3′) and reverse primer (5′–3′)	Product size (bp)
GAPDH (NM_001289726.1)	AGGTCGGTGTGAACGGATTTG and TGTAGACCATGTAGTTGAGGTCA	123
PTEN (NM_008960.2)	ACACCGCCAAATTTAACTGC and TACACCAGTCCGTCCCTTTC	170
IL1 (NM_008361.4)	GCCCATCCTCTGTGACTCAT and AGGCCACAGGTATTTTGTCG	230
IL6 (NM_001314054.1)	AGTTGCCTTCTTGGGACTGA and TCCACGATTTCCCAGAGAAC	159
TNF-*α* (NM_001278601.1)	CGTCAGCCGATTTGCTATCT and CGGACTCCGCAAAGTCTAAG	206
TRAP (NM_001102405.1)	CAGCAGCCAAGGAGGACTAC and ACATAGCCCACACCGTTCTC	190
Cathepsin K (NM_007802.4)	CCAGTGGGAGCTATGGAAGA and AAGTGGTTCATGGCCAGTTC	162
Osteocalcin (OC) (NM_001032298.3)	AAGCAGGAGGGCAATAAGGT and TTTGTAGGCGGTCTTCAAGC	156
ALP (NM_007431.3)	GCTGATCATTCCCACGTTTT and CTGGGCCTGGTAGTTGTTGT	204

## Data Availability

The data used to support the findings of this study are available from the corresponding author upon request.
